# A Case of Bell’s Palsy with an Incidental Finding of a Cerebellopontine Angle Lipoma

**DOI:** 10.7759/cureus.747

**Published:** 2016-08-24

**Authors:** Carlito Lagman, Winward Choy, Seung J Lee, Lawrance K Chung, Timothy T Bui, Isaac Yang, Howard W Goldman

**Affiliations:** 1 Neurosurgery, David Geffen School of Medicine, University of California, Los Angeles; 2 Neurosurgery, Cooper University

**Keywords:** bell palsy, cerebellopontine angle tumor, lipomas

## Abstract

This case report illustrates the potential fallacy of attributing a patient’s symptoms to an incidental finding. Serial imaging of small, asymptomatic cerebellopontine angle (CPA) lipomas is favored. It is imperative to accurately diagnose CPA lipoma on imaging and differentiate it from more common CPA lesions. We herein present a patient with symptoms consistent with Bell’s palsy and an incidental finding of a CPA lipoma. Additionally, we performed a review of the literature for case reports of patients presenting with facial symptoms and diagnosed with a CPA lipoma.

## Introduction

Bell’s palsy is an idiopathic facial paralysis associated with herpes and Lyme disease. Currently, the standard of treatment is anti-viral medications and corticosteroids. Cerebellopontine angle (CPA) lipomas are rare congenital malformations thought to arise from maldifferentiation of the meninx primitiva (a mesenchymal derivative of neural crest cells) [1]. These lesions account for less than one percent of all CPA tumors. Asymptomatic CPA lipomas are often managed with serial surveillance. Most lipomas show a stable size several years after the initial scan. Hearing loss, tinnitus, vertigo, and facial symptoms are common and may be amenable to rehabilitation training and targeted medical therapy. Patients with medically intractable symptoms may be candidates for surgery, but excision at this stage of growth is associated with high surgical morbidity because of adherence to and encasement of critical neurovascular structures. Hearing loss and facial nerve paralysis are the most common complications after surgery. We herein present a case of Bell's palsy with an incidental finding of a CPA lipoma.

## Case presentation

### History and physical examination

A 60-year-old female with a history of diabetes presented to the emergency department after developing acute right-sided facial paralysis upon waking. She also complained of right-sided lagophthalmos, dysgeusia, and inability to keep fluids in her mouth. Two days prior to presentation, she experienced right-sided retroauricular pain that was sharp and radiated to her jaw. On physical exam she was found to have facial asymmetry, decreased sensation to crude touch in the distribution of V2, and reduced hearing, all on the right side. The stroke work-up was negative. A diagnosis of Bell’s palsy was made and the patient was treated accordingly. On repeat examination she complained of pain and paresthesias in the distribution of V2 and V3 (on the right), her facial droop was worse, and she leaned to the right when asked to walk. Involvement of the trigeminal and vestibulocochlear nerves seemed inconsistent with classic Bell’s palsy. The patient underwent further evaluation with magnetic resonance imaging (MRI) of the brain. The patient images and data from previously published literature used for this study are completely de-identified and therefore IRB and/or patient consent was not required for this case report.

### Imaging

**Figure 1:**
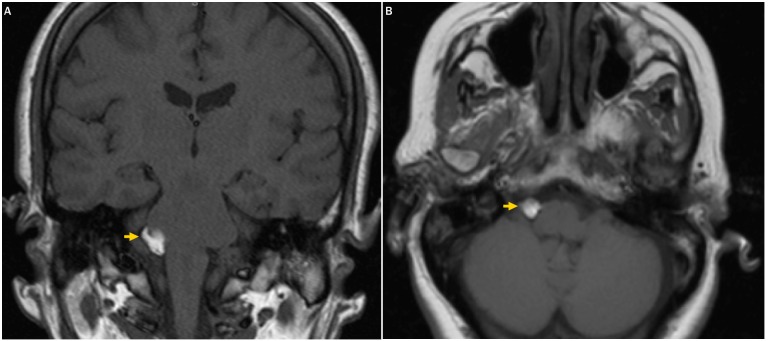
A T1-weighted MRI indicating a right-sided CPA lipoma (A) A coronal T1-weighted MRI and (B) an axial T1-weighted MRI demonstrating a hyperintense mass in the right CPA (yellow arrows) consistent with a lipomatous tumor.

A brain MRI (Figure 1) demonstrated an extra-axial, heterogenous, lobulated T1-hyperintense mass in the right CPA consistent with lipoma. The mass abutted the pons and was noted to have flow voids and linear hypointensities thought to represent vessels and cranial nerves, respectively. At this point the patient's facial symptoms were attributed to the CPA lipoma.

Fat suppression techniques such as short-T1-inversion recovery (STIR; not shown) dampen the signal of lipomas and allow differentiation from other common tumors in the CPA (e.g. vestibular schwannomas and meningiomas). Lipomas are also hypodense and non-enhancing on computed tomography (CT) and display variable signal on T2-weighted imaging. Adherence to brain and encasement of cranial nerves and vessels is common, with erosion of bone and extension into the internal auditory canal (IAC) being less common. Extension into the IAC is associated with poorer prognosis (unpublished data).

## Discussion

The differential diagnosis in our patient included Bell’s palsy, trigeminal neuralgia, stroke, and neoplasm. Bell’s palsy is associated with herpes and Lyme disease, neither of which was present in our patient’s history. Lyme facial palsy is classically bilateral; however, our patient’s facial weakness was unilateral. Trigeminal neuralgia was considered because of the history of paroxysmal pain in the distribution of the trigeminal nerve. However, our patient’s pain was sharp and not as intense as would be expected with trigeminal neuralgia. Further, she lacked any of the classic provocative features associated with trigeminal neuralgia including pain with light touch, chewing, or brushing teeth. Stroke is generally considered in the setting of acute onset facial paralysis and is an upper motor neuron injury; however, our patient’s facial paralysis was consistent with a lower motor neuron injury. Moreover, an initial head CT imaging was negative for stroke. In this case Bell’s palsy diagnosis was favored especially after the patient's condition improved with corticosteroids.

Although CPA lipomas are rare, case reports are abundant in the literature. We reviewed case reports of patients presenting with facial symptoms and diagnosed with a CPA lipoma. An abridged version of our review is summarized in Table 1 (cases from years 2002 to 2015). A supplemental table detailing more cases (from 1859 to 2015) is provided in the Appendix. Tankéré, et al. reported a case of a 56-year-old male who presented with both right-sided Bell’s palsy and a CPA lipoma [2]. Although he recovered, he developed hemifacial spasm. Twelve years after the initial Bell’s palsy diagnosis, the patient was diagnosed with Bell’s palsy on the left side (contralateral). He recovered again; however, an audiometric testing revealed moderate sensorineural hearing loss. He underwent evaluation with MRI, which revealed a 1-mm T1-hyperintense, T2-hypointense lesion at the right cochlear nerve, the latter being a distinguishing feature of lipoma. Adherence of the lipoma to the right cochlear nerve may explain the patient's facial symptoms as the facial and vestibulocochlear nerves are intimately positioned. It is unlikely that the patient's Bell's palsy was related to the lipoma. Moreover, left-sided Bell’s palsy cannot be attributed to a right-sided lipoma. Bell's palsy can present with hearing loss; however, involvement of the right cochlear nerve likely contributed to the patient's hearing loss in this case. We believe the patient's CPA lipoma clinically manifested along with Bell's palsy; however, Bell's palsy was not directly caused by the CPA lipoma.

**Table 1:** Cases of CPA lipoma with associated facial symptoms (2002-2015) R = right; L = left; HFS = hemifacial spasm; TN = trigeminal neuralgia; FNP = facial nerve palsy; CN V = cranial nerve V (trigeminal nerve); hypo = hypodense / hypointense; hyper = hyperintense; Y = yes; Gd = gadolinium contrast enhancement; N = non-enhancing; HL = hearing loss; VT = vertigo; - = not reported; Scan (yrs) = follow-up radiological scan (years after initial diagnosis); S = stable scan; NA = not applicable.

Authors & Year [Ref]	Age (yrs)	Sex	CPA	Facial Symptoms	Size (mm)	CT	T1	T2	Fat-suppressed	Gd	Manage	Outcomes	Complications	Scan (yrs)
Doherty, et al., 2015 [3]	26	F	R	HFS	7	-	Hyper	Iso-hyper	Y	-	Medical	Improved	NA	-
White, et al. 2013 [4]	60	F	R	HFS	12.7	-	-	-	-	-	Surgery	Worsened	HL	S (1.3)
Egemen, et al., 2012 [5]	6	F	L	TN	-	-	Hyper	Hyper	Y	-	Medical	Resolved	NA	-
Shulev, et al., 2011 [6]	48	F	R	TN	-	-	Hyper	-	-	-	Surgery	Improved	Hypoesthesia	-
Marton, et al., 2009 [7]	46	M	L	TN	35	-	Hyper	-	-	-	Surgery	Resolved	None	S (10)
Barajas, et al., 2008 [8]	77	M	L	HFS	-	-	Hyper	-	-	-	Surgery	Resolved	None	-
Schlierter, et al., 2007 [9]	24	M	L	TN	25	-	Hyper	Hyper	Y	N	-	-	-	-
Prasanna, et al., 2003 [10]	22	F	L	FNP	-	-	Hyper	Hyper	-	-	Surgery	Resolved	-	-
Tankéré, et al., 2002 [2]	46	M	R	Bell's palsy, HFS	1	-	Hyper	Hypo	-	-	Observe	Resolved	NA	S (1)

Our patient’s CPA lipoma abuts the pons and encases cranial nerves and vessels (Figure 1), which initially led us to attribute the patient’s facial symptoms to the CPA lipoma. Only after resolution following medicinal treatment was it concluded that the CPA lipoma was incidental and unrelated to the patient’s Bell's palsy. The possibility still exists that the patient's symptoms were attributable to both the CPA lipoma and Bell's palsy. However, the law of parsimony suggests that the CPA lipoma was an incidental finding. To our knowledge, this is the first case of an otherwise asymptomatic CPA lipoma being identified in the setting of ipsilateral Bell’s palsy. On follow-up exam, the patient was asymptomatic and repeat imaging showed a stable lesion.

## Conclusions

In summary, we present a case that illustrates the potential fallacy of attributing a patient’s symptoms to an incidental finding. Furthermore, this case highlights the synthesis of data gathered from clinical assessment and imaging to formulate a differential diagnosis, and ultimately, a therapeutic strategy. Knowledge of subtle radiologic features and ancillary imaging techniques may help one distinguish CPA lipomas from other lesions commonly situated within the CPA and ultimately avoid intrusion into one of the most intricate of neurosurgical chasms: the CPA.

## Appendices

**Table 2:** Cases of CPA with associated facial symptoms (1859-2015) R = right; L = left; HFS = hemifacial spasm; TN = trigeminal neuralgia; FNP = facial nerve palsy; CN V = cranial nerve V (trigeminal nerve); hypo = hypodense / hypointense; hyper = hyperintense; Y = yes; Gd = gadolinium contrast enhancement; N = non-enhancing; HL = hearing loss; VT = vertigo; - = not reported; Scan (yrs) = follow-up radiological scan (years after initial diagnosis); S = stable scan; NA = not applicable.

Reference	Age (yrs)	Sex	CPA	Facial Symptoms	Size (mm)	CT	T1	T2	Fat-suppressed	Gd	Manage	Outcomes	Complications	Scan (yrs)
Doherty CM, Briggs G, Quigley DG, McCarron MO: An unusual cause of hemifacial spasm. Br J Neurosurg. 2015, 29:107-109. 10.3109/02688697.2014.940841	26	F	R	HFS	7	-	Hyper	Iso-hyper	Y	-	Medical	Improved	NA	-
White JR, Carlson ML, Van Gompel JJ, Neff BA, Driscoll CL, Lane JI, Link M: Lipomas of the cerebellopontine angle and internal auditory canal: Primum Non Nocere. Laryngoscope 2013, 123:1531-1536. 10.1002/lary.23882	60	F	R	HFS	12.7	-	-	-	-	-	Surgery	Worsened	HL	S (1.3)
Egemen E, Borcek AO, Karaaslan B, Baykaner MK: Trigeminal neuralgia associated with cerebellopontine angle lipoma in childhood. Pediatr Neurosurg. 2012, 48:306-309. 10.1159/000351550	6	F	L	TN	-	-	Hyper	Hyper	Y	-	Medical	Resolved	NA	-
Shulev Y, Trashin A, Gordienko K: Secondary trigeminal neuralgia in cerebellopontine angle tumors. Skull Base. 2011, 21:287-294. 10.1055/s-0031-1284218	48	F	R	TN	-	-	Hyper	-	-	-	Surgery	Improved	Hypoesthesia	-
Marton E, Basaldella L, Longatti PL: Minimal surgery for a cerebellopontine angle lipoma. J Clin Neurosci. 2009, 16:129-132. 10.1016/j.jocn.2008.01.023	46	M	L	TN	35	-	Hyper	-	-	-	Surgery	Resolved	None	S (10)
Barajas RF, Chi J, Guo L, Barbaro N: Microvascular decompression in hemifacial spasm resulting from a cerebellopontine angle lipoma: case report. Neurosurgery. 2008, 63:E815-816; discussion E816.10.1227/01.NEU.0000325734.30302.97	77	M	L	HFS	-	-	Hyper	-	-	-	Surgery	Resolved	None	-
Schlierter M, Schrey M, Schramm P: Lipoma in cerebellopontine angle. [Article in German] Rofo. 2007, 179:1-3. 10.1055/s-2007-965834	24	M	L	TN	25	-	Hyper	Hyper	Y	N	-	-	-	-
Prasanna AV, Muzumdar DP, Goel A: Lipoma in the region of the jugular foramen. Neurol India. 2003, 51:77-78	22	F	L	FNP	-	-	Hyper	Hyper	-	-	Surgery	Resolved	-	-
Tankere F, Vitte E, Martin-Duverneuil N, Soudant J: Cerebellopontine angle lipomas: report of four cases and review of the literature. Neurosurgery. 2002, 50:626-631	46	M	R	Bell's palsy, HFS	1	-	Hyper	Hypo	-	-	Observe	Resolved	NA	S (1)
Raieli V, Eliseo G, Manfre L, Pandolfi E, Romano M, Eliseo M: Trigeminal neuralgia and cerebellopontine-angle lipoma in a child. Headache. 2001, 41:720-722. 10.1046/j.1526-4610.2001.041007720.x	8	M	R	TN	4	-	Hyper	-	Y	N	Medical	Resolved	NA	S (1)
Alafaci C, Salpietro FM, Puglisi E, Tripodo E, Matalone D, Di Pietro G, Tomasello F: Trigeminal pain caused by a cerebellopontine-angle lipoma. Case report and review of the literature. J Neurosurg Sci. 2001, 45:110-113	16	F	R	TN	20	Hypo	Hyper	-	-	N	Surgery	Resolved	Transient FNP	-
Ruggieri RM, Manfre L, Calbucci F, Piccoli F: Therapeutic considerations in cerebellopontine angle lipomas inducing hemifacial spasm. Neurol Sci. 2000, 21:329-331. 10.1007/s100720070072	45	F	R	HFS	10	-	Hyper	-	Y	-	Medical, surgery	Resolved	R tinnitus, HL	-
Celik SE, Kocaeli H, Cordan T, Bekar A: Trigeminal neuralgia due to cerebellopontine angle lipoma. Case illustration. J Neurosurg. 2000, 92:889. 10.3171/jns.2000.92.5.0889	32	M	L	TN	-	-	Hyper	Hyper	-	-	Surgery	Resolved	FNP, HL	-
Lenthall R, McConachie NS, Jefferson D: Cerebellopontine angle lipoma with an incidental scalp lipoma in a patient with hemifacial spasm. Eur Radiol. 2000, 10:195. 10.1007/s003300050032	60	F	R	HFS	-	-	Hyper	Hyper	-	-	-	-	-	-
Behar PM, Dolan R, Dastur K, Marrangoni AG, Nayak N: Fibrovascular lipoma of the cerebellopontine angle mimicking trigeminal neuralgia. Ear Nose Throat J. 1998, 77:58-60	23	M	R	TN	20	Hypo	Hyper	Hypo	-	N	Surgery	Resolved	HL	-
Heier LA, Comunale JP, Lavyne MH: Sensorineural hearing loss and cerebellopontine angle lesions. Not always an acoustic neuroma--a pictorial essay. Clin Imaging. 1997, 21:213-223. 10.1016/S0899-7071(96)00013-7	21	M	L	TN	-	Hypo	Hyper	-	-	-	Surgery	-	-	-
Kato T, Sawamura Y, Abe H: Trigeminal neuralgia caused by a cerebellopontine-angle lipoma: case report. Surg Neurol. 1995, 44:33-35. 10.1016/0090-3019(95)00056-9	13	F	R	TN	-	Hypo	Hyper	-	-	N	Surgery	Resolved	Transient HL	NA
Inoue T, Maeyama R, Ogawa H: Hemifacial spasm resulting from cerebellopontine angle lipoma: case report. Neurosurgery. 1995, 36:846-850	66	M	R	HFS	15	Hypo	Hyper	Hyper	-	-	Surgery	Worsened	FNP, HL, cerebellar+	S (2)
Ferreira MP, Ferreira NP, Lenhardt R: Lipoma of the cerebellopontine angle. Case reports and literature review. Arq Neuropsiquiatr. 1994, 52:58-63	57	M	R	FNP	18	-	-	-	-	-	Surgery	Resolved	None	-
Ferreira MP, Ferreira NP, Lenhardt R: Lipoma of the cerebellopontine angle. Case reports and literature review. Arq Neuropsiquiatr. 1994, 52:58-63	28	F	L	TN	20	-	-	-	-	-	Surgery	Resolved	HL, FNP	S (3)
Aihara N, Nagai H, Kamiya K, Matsumoto T, Yamashita N: Cerebellopontine angle lipoma--case report. [Article in Japanese] No To Shinkei. 1993, 36:846-850	47	F	R	TN	-	Hypo	Hyper	-	-	-	Surgery	-	-	-
Nakao S, Yamamoto T, Fukumitsu T, Ban S, Motozaki T, Sato S, Otsuka S, Nakatsu S, Tabuchi T, Saiwai S: Cerebellopontine angle lipoma. Case report. Neurol Med Chir (Tokyo). 1998, 28:1113-1118. http://www.ncbi.nlm.nih.gov/pubmed/?term=2466216	48	M	L	TN	-	Hypo	Hyper	Hyper	Y	N	Surgery	Unimproved	Diplopia, FNP, HL	Stable (.4)
Sprik C, Wirtschafter JD: Hemifacial spasm due to intracranial tumor. An international survey of botulinum toxin investigators. Ophthalmology. 1988, 95:1042-1045. doi:10.1016/S0161-6420(88)33044-7	26	M	R	HFS	-	-	-	-	-	-	Observe	-	-	-
Levin JM, Lee JE: Hemifacial spasm due to cerebellopontine angle lipoma: case report. Neurology. 1987, 37:337-339	22	M	L	HFS	-	-	-	-	-	-	Surgery	Resolved	Transient HL, tinnitus, FNP	-
Christensen WN, Long DM, Epstein JI: Cerebellopontine angle lipoma. Hum Pathol. 1986, 17:739-743	55	F	L	FNP	-	-	-	-	-	-	Surgery	Worsened	-	-
Christensen WN, Long DM, Epstein JI: Cerebellopontine angle lipoma. Hum Pathol. 1986, 17:739-743	28	M	L	FNP	-	-	-	-	-	-	Surgery	Resolved	HL, VT	-
Rosenbloom SB, Carson BS, Wang H, Rosenbaum AE, Udvarhelyi GB: Cerebellopontine angle lipoma. Surg Neurol. 1985, 23:134-138	28	M	L	CN V paresis	-	-	-	-	-	-	Surgery	Improved	HL	-
Graves VB, Schemm GW: Clinical characteristics and CT findings in lipoma of the cerebellopontine angle. Case report. J Neurosurg. 1982, 57:839-841. 10.3171/jns.1982.57.6.0839	26	M	L	TN	15	Hypo	-	-	-	N	Surgery	Resolved	HL	-
Budka H: Intracranial lipomatous hamartomas (intracranial "lipomas"). A study of 13 cases including combinations with medulloblastoma, coloid and epidermoid cysts, angiomatosis and other malformations. Acta Neuropathol. 1974, 28:205-222	26	F	R	TN	-	-	-	-	-	-	Surgery	Resolved	-	-
